# Efficacy of atosiban for repeated embryo implantation failure: A systematic review and meta-analysis

**DOI:** 10.3389/fendo.2023.1161707

**Published:** 2023-03-23

**Authors:** Ruxin Wang, Haixia Huang, Yong Tan, Guicheng Xia

**Affiliations:** ^1^ First Clinical Medical College, Nanjing University of Chinese Medicine, Nanjing, Jiangsu, China; ^2^ Department of Reproductive Medicine, Affiliated Hospital of Nanjing University of Chinese Medicine, Nanjing, Jiangsu, China

**Keywords:** atosiban, repeated embryo implantation failure, *in vitro* fertilization-embryo transfer (IVF-ET), meta-analysis, clinical pregnancy rate (CPR)

## Abstract

**Background:**

Repeated embryo implantation failure (RIF) posed a significant challenge in assisted reproduction. Evidence of its therapeutic effectiveness including atosiban used around embryo transfer to improve pregnancy outcomes in RIF patients undergoing *in vitro* fertilization-embryo transfer (IVF-ET) remained inconsistent. This study aimed to explore the efficacy of atosiban on pregnancy outcomes of patients with RIF who received IVF-ET.

**Methods:**

The research was designed using the PICOS format. A systematic search of four English databases, PubMed, EMBASE, Web of Science, Cochrane Library, and one Chinse database, China National Knowledge Infrastructure (CNKI) was conducted. The time range was from inception to December 10, 2022. Then trials comparing the efficacy of atosiban and control group on pregnancy outcomes in RIF patients who receive IVF-ET were included. Subgroup analysis and sensitivity analysis were performed to reduce the influence of heterogeneity between included studies. Risk ratio (RR) and 95% confidence interval (CI) were calculated. The main outcome measure was clinical pregnancy rate (CPR). For the analyses, StataMP 17.0 (Stata Corporation, USA) was used.

**Results:**

Two prospective randomized controlled trials (RCTs), one prospective cohort study and four retrospective cohort studies were included. Our results showed that atosiban was associated with higher clinical pregnancy rate (RR=1.54, 95% CI: 1.365–1.735, *P* < 0.001, I^2 =^ 0.0%). The results of subgroup analysis based on study types (prospective randomized controlled clinical trial, retrospective cohort study and prospective cohort study) showed that in all types of studies, CPR of atosiban group was significantly higher than controlled group. The results of subgroup analysis based upon the diagnostic criteria of number of previous embryo transfer failures showed that the intervention of atosiban improved the CPR whether in participants with 2 previous ET failures or in participants with 3 previous ET failures. Nevertheless, the incidence of ectopic pregnancy, multiple pregnancy, and miscarriages were not significantly different between the case and control groups.

**Conclusion:**

For women who are undergoing IVF-ET and have experienced repeated embryo implantation failure, atosiban may be an important factor in enhancing pregnancy outcomes. To confirm this conclusion, more thorough, prospective randomized controlled studies of sizable sample sizes with well design are required.

## 1 Introduction

One of the most crucial stages in reproduction, embryo implantation is the process by which the embryo connects to the luminal surface of the endometrium, moves through the luminal epithelium, and infiltrates the deep layer to become fixed in the deeper layer (1). In assisted reproductive technology (ART), ultrasonographic evidence of an intrauterine gestational sac suggests that the progress of implantation is completed successfully which necessitates a competent blastocyst, a receptive endometrium and synchronous communication between the maternal and embryonic tissues (2). Embryo abnormalities, poor endometrial receptivity as well as insufficient interaction between embryo and maternal endometrium can lead to implantation failure.

Repeated embryo implantation failure (RIF) is an unsolved and challenging technical problem during *in vitro* fertilization-embryo transfer (IVF-ET). At present, no standard definition has been established for the total number of transferred embryos or the number of failed cycles. It is however accepted that RIF can be considered as the inability to successfully achieve a clinical pregnancy after receiving embryo transfers (ETs) of high-quality embryo three or more times or ≥10 embryos transferred at different times with the precise numbers of transfers to be chosen by each different reproductive medical centers (3). Accordingly, there are different definitions for RIF in different centers practicing IVF. It is also well-accepted that failure of pregnancy after two or more embryo transfer cycles for individuals constitute RIF (4, 5). Those failures may bring these infertile couples tremendous mental and economic pressure (6).

At present, more attention has been attracted regarding how to improve pregnancy outcomes of patients experienced RIF. Traditionally, the quality of embryo has been considered as the main cause for RIF. Indeed, impaired uterine receptivity was thought to be one of the main causes of treatment failure when high-quality embryos were transplanted (7). Generally, structural uterus abnormal including uterine congenital abnormalities and acquired diseases (8), thickness of endometrium (9, 10), chronic endometritis (11), endometrial perfusion (12) and uterine peristalsis (13) may impact on endometrial receptivity and thus embryo implantation. Previous studies proved that in fresh and frozen-thawed embryo transfer cycles, uterine peristalsis had a significant impact in embryo mobility and implantation (14) and was even associated with the clinical pregnancy outcome (15, 16). Such as, with an increase in uterine peristalsis, the rates of implantation, clinical pregnancy, and continued pregnancy gradually reduced (17). Excessive uterine peristalsis could move the implanted embryo out of the uterus. Thus, uterine peristalsis was considered as a potential triggers on decreasing implantation rates in ART cycles. In contrast, uterine peristalsis has been neglected in diagnostic measures, and it has not been demonstrated that treatments around ET like beta agonists or non-steroid anti-inflammatory medications (NSAIDs) are beneficial in decreasing uterine peristalsis (18).

Atosiban, a vasopressin V1a and oxytocin receptor antagonist, was selected as the treatment for preterm labour by reducing uterine peristalsis (19). The application of atosiban in IVF that may decrease uterine peristalsis to improve uterine receptivity during ET was first reported by Pierzynski et al. in 2007 (20). In recent years, many clinical studies evaluating more outcome measures, such as clinical pregnancy, live birth, miscarriage, multiple pregnancy, implantation and ectopic pregnancy rates has been conducted on this issue (21–26). The effectiveness of atosiban intervention in IVF-ET still remained controversial and ambiguous based on the published evidence.

Taking into account the difficulties in treating RIF, atosiban is being applied to reduce uterine peristalsis as an adjuvant to IVF in RIF-affected women. It is necessary to provide objective evidence on the application of atosiban on RIF patients who undergo IVF-ET. This systematic review and meta-analysis were designed to investigate effects of atosiban on IVF-ET-assisted pregnancy outcomes in women with RIF.

## 2 Materials and methods

The PRISMA (Preferred Reporting Items for Systematic Reviews and Meta-analyses) statement was followed when conducting this study (27).

### 2.1 Protocol registration

This review protocol has been registered in the PROSPERO International Prospective Register of Systematic Reviews (Registration Number: CRD42022382312).

### 2.2 PICOS

This study was designed as “PICOS” principle (population, intervention, comparison, outcome and study design). Population: Participants undergoing IVF-ET who had experienced RIF were included. Intervention and comparison: Trials comparing the use of atosiban around ET versus either no treatment or a placebo were eligible for inclusion. Outcome: Trials with the following outcomes were included: positive pregnancy test rate, clinical pregnancy rate, live birth rate, implantation rate, miscarriage rate, multiple pregnancy rate and ectopic pregnancy rate. Study design: Published clinical research (observational studies/clinical trials) were eligible for inclusion.

### 2.3 Literature search

Electronic databases were systematically searched to find all pertinent studies, including PubMed, EMBASE, Web of Science, Cochrane Library, and China National Knowledge Infrastructure (CNKI) by two authors (RXW and HXH) from inception to December 10, 2022. The databases were searched using the following search terms:(*in vitro* fertilization-embryo transfer [Title/Abstract]) OR (IVF-ET [Title/Abstract]) OR (repeated embryo implantation failure [Title/Abstract])) OR (recurrent embryo implantation failure [Title/Abstract]) OR (RIF [Title/Abstract]) OR (intracytoplasmic sperm injection [Title/Abstract]) OR (ICSI [Title/Abstract]) OR (assisted reproductive techniques [Title/Abstract]) OR (ART [Title/Abstract]) OR (*in vitro* fertilization [Title/Abstract]) OR (IVF [Title/Abstract]) OR (embryo transfer [Title/Abstract]) OR (ET [Title/Abstract]) AND (atosiban [Title/Abstract]).

### 2.4 Eligibility criteria

All clinical research (observational studies/clinical trials) examining impacts of atosiban on patients with RIF undergoing IVF-ET were included in this review.

Inclusion criteria (1): The studies involved patients undergoing IVF-ET who had experienced RIF. RIF was defined as ≥2 failed ET cycles (2). The case group was composed of patients treated with atosiban around ET. (3) Patients in the control group underwent either no treatment or a placebo. (4) Confirmed pregnancy outcomes were reported, including at least the following three outcome indicators: implantation rate, clinical pregnancy rate, miscarriage rate. (5) The raw data were available in the articles.

Exclusion criteria: (1) Animal experiments. (2) No usable data was provided. (3) Studies that did not have a control group or a full text available. (4) Reviews and case reports

### 2.5 Study selection and data extraction

Two writers (RXW and HXH) independently selected the studies and extracted the data. All articles from the electronic searches, including abstracts, were evaluated. Citations that met the criteria for inclusion were obtained. A PRISMA flow diagram was created to display the search results as well as the number of trials that were included and excluded. For all included studies, characteristics were summarized in tables, including authors’ names, title, year of publication, number of patients, year of patients, type of study, RIF diagnostic criteria, type of interventions, controlled ovarian stimulation (COS) protocol, ET protocol and outcomes.

### 2.6 Evaluation of bias risk and methodological quality in included studies

The bias risks of included RCTs were evaluated by the criteria of the Cochrane’ risk of bias assessment tool (28). Two evaluators evaluated the reports in terms of the following items independently, assigning scores of “high” “low” and “unclear”: (1) Random sequence generation. (2) Allocation concealment. (3) Blinding of participants and personnel. (4) Blinding of outcome assessment. (5) Incomplete outcome data. (6) Selective reporting. (7) Other sources of bias.

The Newcastle-Ottawa Scale (NOS) was used to evaluate the quality of the cohort studies that were included (29). The NOS checklist involves 3 quality parameters: (1) Selected population. (2) Comparability of groups. (3) Assessment of either the exposure or outcome of interest for case-control or cohort studies. Each study received a grade ranging from 0 to 9. High quality studies were those whose scores were greater than or equal to 7 (30–32).

### 2.7 Synthesis and analysis of information

Using both fixed and random effects models, the pooled risk ratio (RR) with 95% confidence interval (CI) were derived from individual research (33). The results of the meta-analyses were graphically displayed using the forest plot. Statistics were deemed significant at *P* < 0.05. Cochrans *Q* and the *I^2^* statistic were employed to calculate the degree of statistical heterogeneity. A value of 0% indicated no heterogeneity, while values greater than 50% indicated significant heterogeneity (34). When the heterogeneity was less than 50%, a fixed-effect model was chosen; otherwise, a random effects model was chosen. The subgroup analyses were conducted based on study types and the diagnostic criteria of number of previous embryo transfer failures to explore whether the type of the study and the diagnostic criteria influenced the results of meta-analysis. Moreover, to assess the stability of the results, sensitivity analyses were performed. For the analyses, StataMP 17.0 (Stata Corporation, USA) was used. The potential publication bias was graphically evaluated using the Egger’s test (*P* > 0.05).

### 2.8 Definition of outcomes

The clinical pregnancy rate (CPR) was the main outcome indicator of this study; secondary outcome indicators included positive pregnancy test rate (PPTR), live birth rate (LBR), Implantation rate (IR), miscarriage rate (MR), multiple pregnancy rate (MPR), ectopic pregnancy rate (EPR). Clinical pregnancy was verified when the heartbeat of the fetal sac in the uterus was confirmed by ultrasonography. A successful delivery of live-born baby (after 20 weeks of gestation) was defined as a live birth. The implantation rate was identified as the percentage of transferred embryos that successfully underwent implantation, that was the total number of pregnancy sacs per total number of embryos transferred. Miscarriage was commonly defined as a pregnancy loss prior to viability. A pregnancy with more than one fetus was considered as multiple pregnancy. Ectopic pregnancy meant that a fertilized egg implanted outside the main cavity of the uterus.

## 3 Results

### 3.1 Description of studies

**Figure 1:**
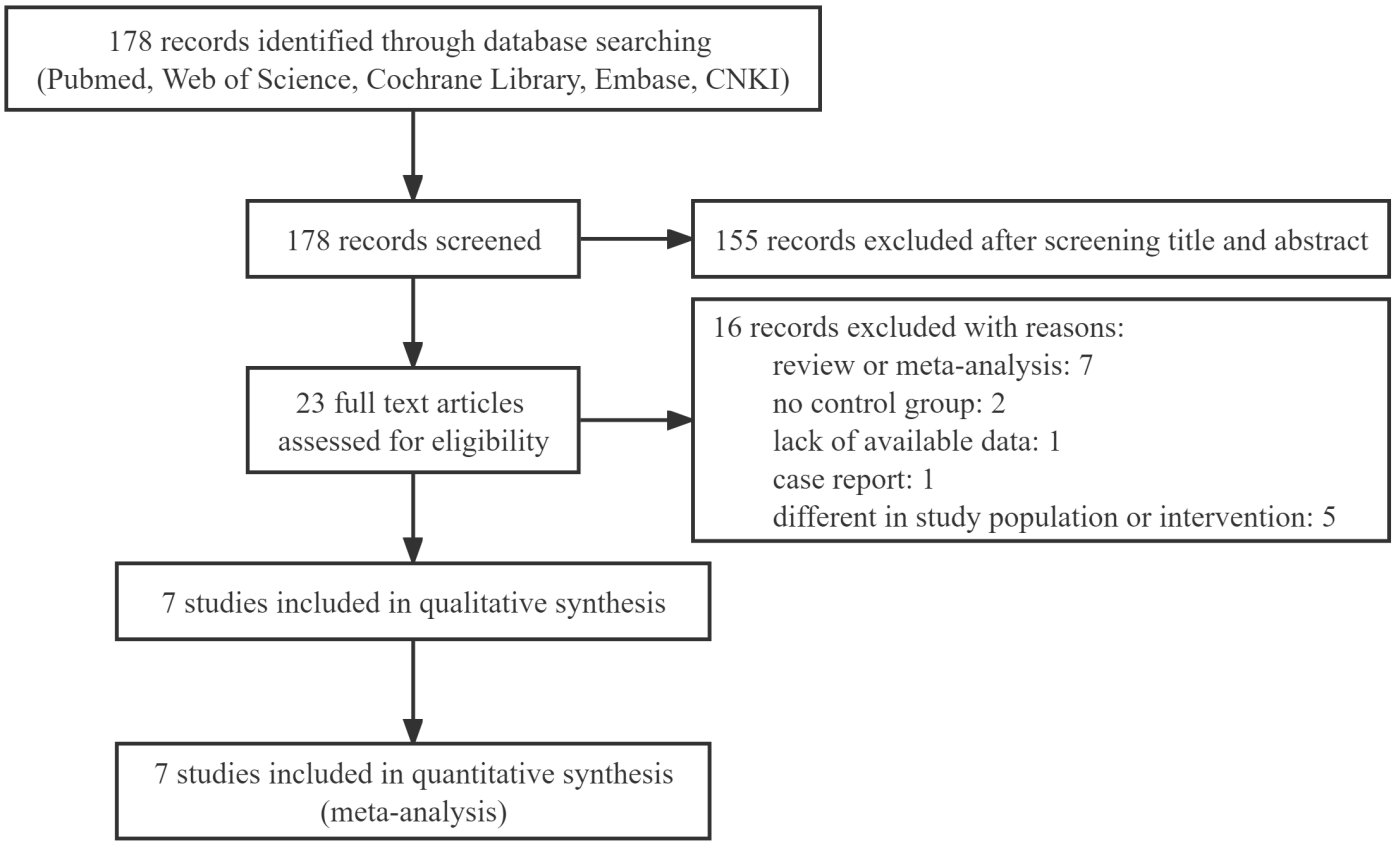
PRISMA flow diagram of study selection process.

This review retrieved 178 relevant records. An assessment of the titles and abstracts revealed 23 records that would be acceptable for inclusion. Among them, due to obvious ineligibility, 16 records were excluded, including meta-analyses, reviews, and case reports, no control group, a lack of available data and different in study population and intervention. Finally, the meta-analysis included 7 studies. A PRISMA flow diagram depicted the selection process in detail (**Figure 1**).

**Table 1** showed the characteristics of the selected studies in detail. Two of the studies that were included were prospective, randomized, double-blind clinical trials (35, 40), one was prospective cohort study (38) and four were retrospective cohort studies (36, 37, 39, 41). 1958 women, 903 participants in case groups, and 1055 participants in control groups were all part of single-center investigations. All patients received IVF or ICSI treatment, with fresh ET or frozen-thawed ET. Three research (39–41) examined the impact of atosiban on patients who underwent two or more ET cycles, while three studies (35–37) involved patients who underwent three or more cycles. One study (38) divided patients into four subgroups based on the number of previous ETs (patients undergoing ET for the first/second/third or more time). Our study included patients who undergoing the third and more than the third ET. The prospective cohort study by He et al. (38) also measured uterine contractions and serum oxytocin (OT), Prostaglandin F2α (PGF2α) level. The number of transfer cycles and serum OT levels were found to positively correlate with uterine contractions, and patients who had higher uterine contractions (43.1 wave/min) were more likely to be the RIFs and benefited more from atosiban treatment.In all studies, atosiban was given intravenously. In six studies, the dose of atosiban was 6.75mg, while the dose was 37.5mg in the study by Zhang Yue et al. (39). The study by Chou et al. (41) investigated if the different methods of atosiban use had an effect on efficacy. Patients who received atosiban were divided into two groups by the usage, a single bolus dose (6.75 mg, 0.9 mL/vial) before ET or a bolus dose of 6.75 mg atosiban followed by a 3-hour infusion at 18 mg/hr after ET. Results indicated that the clinical pregnancy rate and the implantation rate were significantly higher in the group who received a single bolus dose of atosiban before ET. That may suggest a better usage of atosiban.

**Table 1:** Characteristics of included studies in the review.

Study	Design	Number of patients	Age (years)	RIF diagnostic criteria	Intervention	COS protocol	ET protocol	Outcome
Tang et al. (2022) (35)	Prospective, randomized, double-blind controlled clinical trial	194 Atosiban group: 97 Placebo group:97	less than 40	At least 3 fresh or frozen-thawed transfer cycles failure of four good-quality embryos	Atosiban group: a bolus intravenous dose of 6.75 mg atosiban 30 min before ET; Placebo group: normal saline infusion for the same duration	GnRH agonist protocol; GnRH antagonist protocol	Fresh embryo transfer	PPTR;CPR; OPR; LBR; MR; MPR; IR; EPR; CAR
Li et al. (2021) (36)	Retrospective cohort study	388 Atosiban group: 193 Control group: 195	20 – 39	At least 3 previous embryo implantation failure (including fresh cycle and frozen-thawed cycle)	Atosiban group:a bolus intravenous dose of 6.75 mg atosiban 30 min before ET; Control group: no treatment	N/A	Artificial FET cycles	PPTR; CPR; IR; MR; EPR
Liu et al. (2017) (37)	Retrospective cohort study	262 Atosiban group: 97 Control group: 168	less than 40	At least 3 ET failures (including fresh and frozen cycles); or at least one good-quality embryos in a minimum of 10 embryos	Atosiban group:a bolus intravenous dose of 6.75 mg atosiban 30 min before ET; Control group: no treatment	N/A	Natural FET cycles; Artificial FET cycles; Ovulation induction FET cycles	CPR; IR; MR;MPR; EPR; LBR
He et al. (2016) (38)	Prospective cohort study	536 1st ET: 178 2nd ETs: 151 3rd ETs: 119 >3 ETs: 88	20 – 45	With 3 or more transfer cycles	Treatment group: a bolus intravenous dose of 6.75 mg atosiban 30 min before ET; Control group: No treatment	N/A	Natural FET cycles; Artificial FET cycles	PPTR; IR; CPR; MR
Zhang et al. (2014) (39)	Retrospective cohort study	240 Atosiban group: 120 Control group:120	22 – 39	≥2 embryo transfers; the total number of transplanted embryos ≥6; at least one high-quality embryo in each transfer cycle	Atosiban group: intravenous administration of atosiban about 1 hour before the transfer with a bolus dose of 37.5 mg during one hour; Control group: no treatment	N/A	Natural FET cycles; Artificial FET cycles;	IR; CPR; MR; MPR; EPR; LBR
Jiang et al. (2014) (40)	Prospective, randomized controlled clinical trial	188 Atosiban group: 84 Comtrol group: 104	20 – 40	>2 transfer cycles failure of good-quality embryos	Atosiban group: a bolus intravenous dose of 6.75 mg atosiban 30 min before ET; Control group: no treatment	Ultra Long GnRH agonist protocol; Long GnRH agonist protocol; Short GnRH agonist protocol; GnRH antagonist protocol ; Mild stimulation protocol	Natural FET cycles; Artificial FET cycles;	CPR; IR;MPR; MR
Chou et al. (2011) (41)	Retrospective cohort study	150 Group 1: 80 Group 2: 40 Group 3: 30	Group 1: 34.8±3.76 Group 2: 34.63±4.21 Group 3: 34.63±4.21	2 or more previous IVF failures after the transfer of good-quality embryos	Group 1: no treatment; Group 2: a single bolus dose (6.75 mg, 0.9 mL/vial) of atosiban intravenously before ET; Group 3: a bolus dose of 6.75 mg atosiban at 18 mg/hr for 3 hours	Long luteal-phase GnRH agonist protocol; Short GnRH agonist protocol; GnRH antagonist protocol	Fresh embryo transfer	IR, CPR, MR,LBR,MPR

PPTR, Positive pregnancy test rate; CPR, Clinical pregnancy rate; OPR, Ongoing pregnancy rate; IR, Implantation rate; LBR, Live birth rate; MR, Miscarriage rate; MPR, Multiple pregnancy rate; EPR, Ectopic pregnancy rate; CAR, Congenital abnormality rate; N/A, Not applicable.

### 3.2 Risk of bias assessment and quality evaluation

Based on various quality domains of the Cochrane Collaboration tool, the risks of bias of the included RCTs were showed in **Table 2**. One of the RCTs (35) was at low risk of bias for method of randomization, allocation concealment, performance bias, detection bias, attrition bias and reporting bias. In the study (40), methods for random sequence generation and random allocation concealment were at high risk of selection bias. Patients in that study were randomized according to the ET day and informed consent and there was no blinding in this study. Since the outcomes of implantation, clinical pregnancy, multiple pregnancies, and miscarriage are all evaluated objectively by serum human chorionic gonadotrophin (HCG) detection and ultrasound scan, it was improbable that the assessment of pregnancy outcome would be subjective. Thus we believed that detection bias of all studies were at a low risk.

**Table 2:** Bias risks of the included RCT.

Study, year	Domain 1	Domain 2	Domain 3	Domain 4	Domain 5	Domain 6	Domain 7
Tang et al. (2022) (35)	Low	Low	Low	Low	Low	Low	Low
Jiang et al. (2014) (40)	High	High	High	Low	Low	Low	Low

Bias risk was determined using the Cochrane risk of bias tool: 1: Random sequence generation (selection bias); 2: Allocation concealment (selection bias); 3: Blinding of participants and personnel (performance bias); 4: Blinding of outcome assessment (detection bias); 5: Incomplete outcome data (attrition bias); 6: Selective reporting (reporting bias); 7: Other sources of bias (other bias).

The Newcastle-Ottawa Scale (NOS) was used to assess the quality of the included cohort studies in **Table 3**. The NOS scores of included cohort studies ≥ 7 points were considered to be of high quality. None of the included studies mentioned non-response rate.

**Table 3:** Bias risks of the included cohort studies.

Study, year	Quality evaluation	Selection				Comparability	Exposure		
		Case definition	Representa -tiveness	Selection of controls	Definition of controls	Comparability	Ascertainment of exposure	Same method	Non-Response rate
Li et al. (2021) (36)	8	1	1	1	1	2	1	1	0
Liu et al. (2017) (37)	7	1	1	1	1	1	1	1	0
He et al. (2016) (38)	8	1	1	1	1	2	1	1	0
Zhang et al. (2014) (39)	7	1	1	1	1	1	1	1	0
Chou et al. (2011) (41)	8	1	1	1	1	2	1	1	0

**Figure 2:**
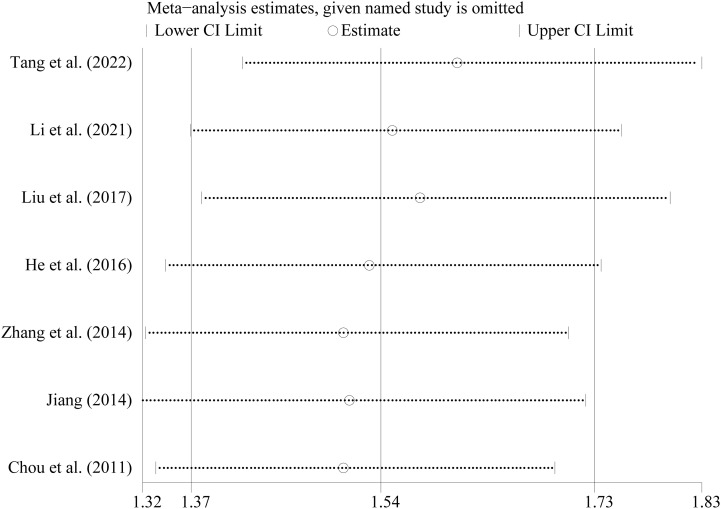
Sensitivity analysis of the included studies The abscissa refer to the RR and the ordinate represented each study and year.

Considering the various study designs that were used, we performed a sensitivity analysis based on the main outcome indicator (clinical pregnancy rate) which showed a stable and reliable outcome. The pooled analysis was not significantly impacted by any of the studies. Regardless of any study excluded, the results remained statistically significant (RR=1.54, 95% CI: 1.37–1.73) (**Figure 2**). The Egger regression asymmetry test revealed no statistically significant publication bias (Egger’s test; t = 0.76, *P* = 0.482).

### 3.3 Outcome measures

#### 3.3.1 Positive pregnancy test rate

The rates of PPTR were examined in three studies. A total of 603 participants were included. The combined PPTR was 55.7% in the atosiban group and 42.0% in the control group. Treatment with atosiban strongly improved positive pregnancy test rate by the fixed-effects model and the RR was 1.32(95% CI: 1.12 – 1.56, *P*=0.001, I^2 ^= 36.4%) (**Table 4**; **Supplemental Figure 1**).

**Table 4:** Meta-analysis of all studies comparing pregnancy outcomes between case and control groups in patients with RIF.

Pregnancy outcomes	Number of studies	Number of participants	Positive/total in case group	Positive/total in control group	Risk Ratio	95% Confidence Interval	I^2^	*P*	Analysis model
Positive pregnancy test rate	3	1118	55.7% (165/296)	42.0% (129/307)	1.32	1.12 - 1.56	36.4%	0.001	fixed-effects
Clinical pregnancy rate	7	1958	52.4% (348/664)	34.7% (270/779)	1.54	1.37 - 1.74	0.0%	<0.001	fixed-effects
Live birth rate	4	846	40.4% (154/381)	26.7% (124/465)	1.58	1.18 – 2.11	49.8%	0.002	random-effects
Implantation rate	7	1958	34.2% (460/1345)	22.8% (341/1496)	1.54	1.37 – 1.74	15.9%	<0.001	fixed-effects
Miscarriage rate	7	1958	11.7% (46/392)	12.0% (40/334)	0.94	0.63 – 1.39	0.0%	0.747	fixed-effects
Multiple pregnancy rate	5	1034	26.3% (65/247)	19.9% (40/201)	1.26	0.88 – 1.79	0.0%	0.212	fixed-effects
Ectopic pregnancy rate	4	1084	2.8% (6/214)	4.5% (8/179)	0.64	0.23 – 1.83	0.0%	0.409	fixed-effects

#### 3.3.2 Clinical pregnancy rate

Results showed that atosiban significantly improved CPRs in all included studies in women with RIF, according to the fixed effect forest plot (RR=1.54, 95% CI: 1.37–1.74, *P*<0.001, I^2^ = 0.0%) (**Table 4**; **Supplemental Figure 2**).

**Figure 3:**
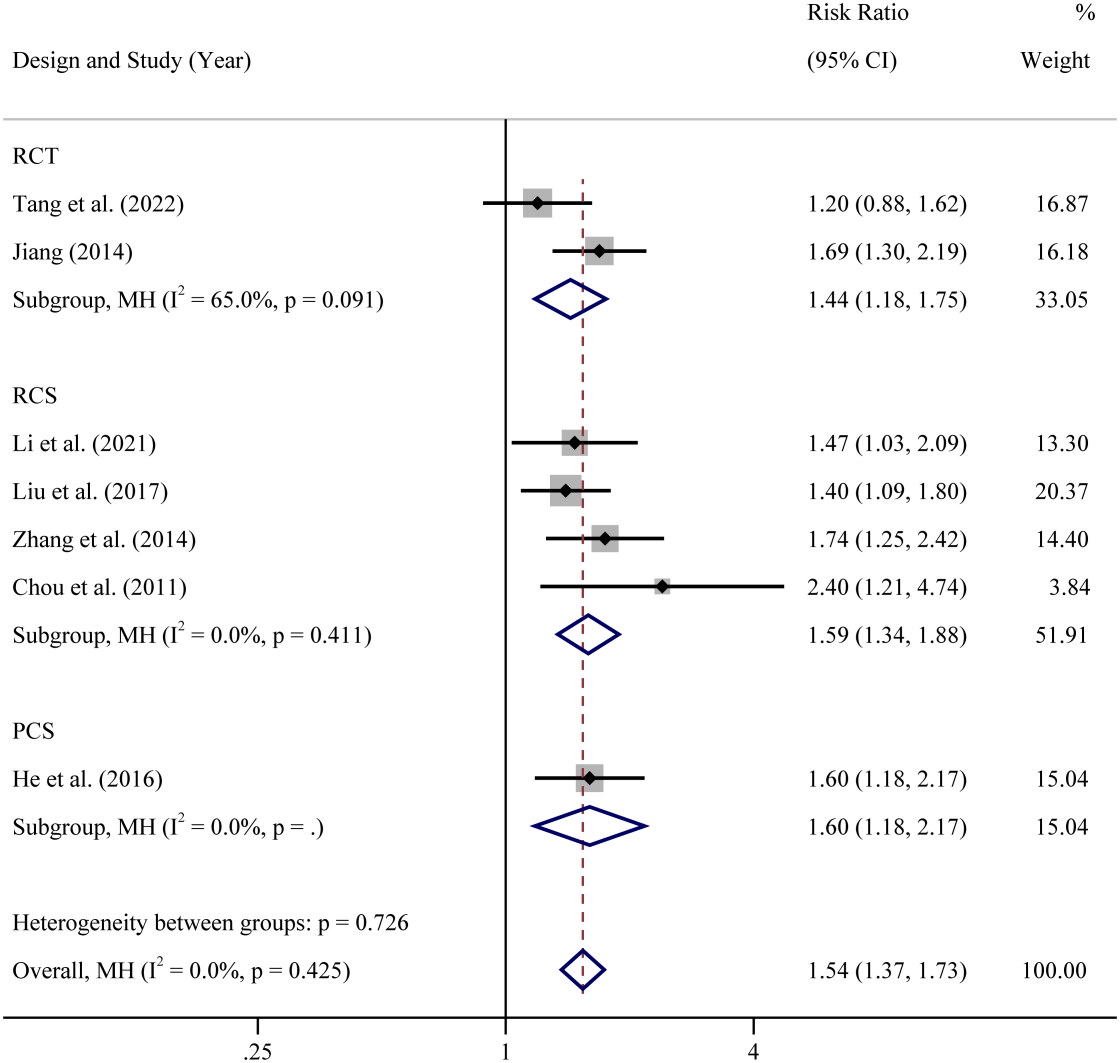
Subgroup analysis based on study design of clinical pregnancy rate RCT: In view of the different designs of the included studies, RCS: In view of the different designs of the included studies, PCS: In view of the different designs of the included studies.

In view of the different types of the included researches, subgroup analysis was performed based on study types (prospective randomized controlled clinical trial, retrospective cohort study and prospective cohort study). The results showed that in all types of studies, whether RCTs or cohort studies, CPR of atosiban group was significantly higher than controlled group (**Figure 3**). However, there was a subgroup of RCTs with high level of heterogeneity (65%). The heterogeneity was complicated and may due to many factors. First, age range, sample size, body mass index, sex hormone level and other base line information were different or unknown. Second, low quality of study, such as blindness, methods for random sequence generation and random allocation concealment, may affect the credibility of the results. Third, the control measures were different in two studies. One is no treatment while the other one is placebo.

**Figure 4:**
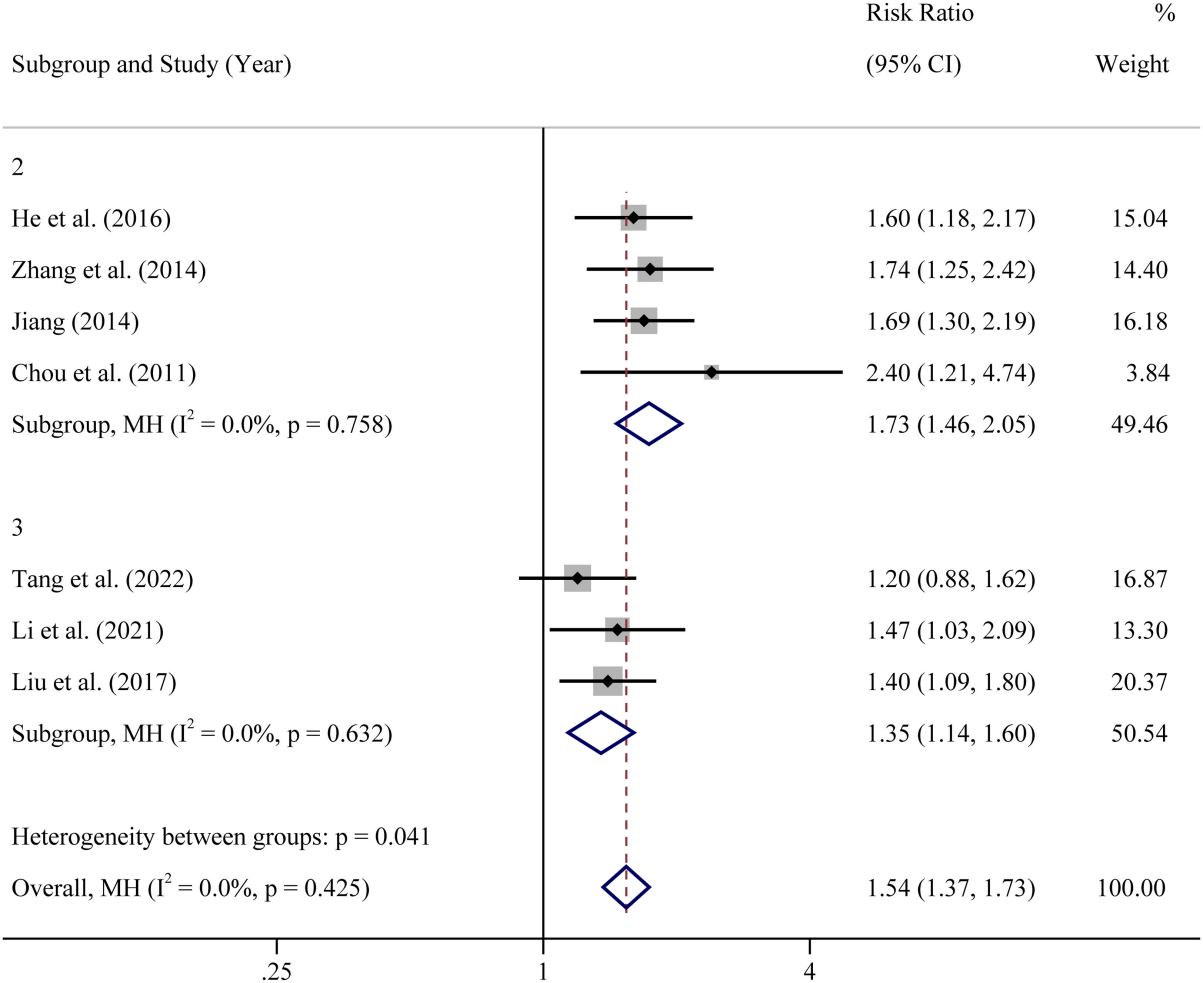
Subgroup analysis based on RIF diagnostic criteria of clinical pregnancy rate.

The diagnostic criterias of RIF were somewhat different among the included studies. In four studies, women with 2 or more transfer cycle failures of good-quality embryos were selected as participants. In other three studies, women with 3 or more transfer cycle failures were selected. Hence, subgroup analysis was undertaken based upon the diagnostic criteria of number of previous embryo transfer failures. The results showed that the intervention of atosiban improved the CPR whether in participants with 2 previous ET failures or in participants with 3 previous ET failures (**Figure 4**).

#### 3.3.3 Live birth rate

LBR was selected as one of two crucial outcomes for IVF/ICSI by ESHRE in their 2019 guideline on ovarian stimulation (42). Four studies examined the effects of atosiban on the LBRs in women with RIF, including 846 participants. Results of the meta-analysis indicated that the administration of atosiban was associated with a higher LBR in RIF patients who receive IVF-ET (RR=1.58, 95% CI: 1.18 – 2.11, *P*=0.002, I^2^ = 49.8%) (**Table 4**; **Supplemental Figure 3**).

#### 3.3.4 Implantation rate

All included studies assessed the implantation rate. We pooled the data and discovered that application of atosiban significantly increased the implantation rates (RR=1.54, 95% CI: 1.37–1.74, *P*<0.001, I^2^ = 15.9%) (**Table 4**; **Supplemental Figure 4**).

#### 3.3.5 Miscarriage rate

The comparison of MRs for 726 patients was conducted in seven related studies. Miscarriage occurred in 46 of 392 (11.7%) patients in the atosiban group and in 40 of 334 (12.0%) patients in the control group. Regarding the rates of miscarriage, no significant difference was found between the two groups; the RR was 0.94 (95% CI: 0.63–1.39, *P*=0.747, I^2^ = 0.0%) in the fixed-effects model (**Table 4**; **Supplemental Figure 5**).

#### 3.3.6 Multiple pregnancy rate

For the MPR, we merged the outcomes of five studies with 448 participants. The combined MPR was 26.3% in the atosiban group and 19.9% in the control group. Results of analysis indicated that there was no significant difference between the atosiban group and the control group in MPR (RR=1.26, 95% CI: 0.88–1.79, *P*=0.212, I^2^ = 0.0%) ((**Table 4**; **Supplemental Figure 6**).

#### 3.3.7 Ectopic pregnancy rate

Four studies with 393 participants focused on the EPR. In the atosiban group, the total EPR was 2.8%, whereas in the control group, it was 4.5%. Results of the meta-analysis showed that differences in the EPR for intervention and control groups were not statistically significant (RR=0.64, 95% CI: 0.23–1.83, *P*=0.409, I^2^ = 0.0%) (**Table 4**; **Supplemental Figure 7**).

## 4 Discussion

This study aimed to learn more about the effects of atosiban medication in patients with RIF undergoing IVF-ET by comparing larger samples of atosiban intervention patients and control patients. In this paper, results indicated that atosiban was associated with a higher positive pregnancy test rate, a higher clinical pregnancy rate, a higher live birth rate and a higher implantation rate. The outcomes demonstrated that there was no discernible difference in the rates of miscarriage, multiple pregnancy, or ectopic pregnancy between the atosiban intervention and control groups.

There were some previous systematic reviews and meta-analyses (43–45) published about the use of atosiban in IVF treatment, but not for patients with RIF. They concluded that in the majority of women who experienced IVF, atosiban might only have a little impact on pregnancy outcomes. However, based on the results of this study, atosiban has significant therapeutic effects on patients with RIF.

Maternal age played a crucial role in the success of IVF and it was one of risk factors for RIF. It was reported that oocyte yield, blastocyst formation and endometrial thickness all decreased in patients over 35 years of age (46). Body mass index (BMI) (47), psychological stress (48), alcohol abuse and smoking (49) were also risk factors for RIF. Embryo and endometrial synchrony was under influence of many factors, such as embryonic and parental genetics, anatomical factors, maternal immune system, endocrine milieu, hematologic factors and reproductive tract microbiome (50). Besides, one of the essential elements of uterine receptivity, uterine contractions, played an important role in embryo implantation (51). Uterine contractions were caused by the synthesis of oxytocin, which was strongly influenced by estradiol (E_2_) level (52). By enhancing the oxytocin receptor gene expression in the uterus, a high amount of E_2_ strengthened the effects of oxytocin, leading to uterine contractions even without pregnancy (53). Also, a high level of E_2_ may induce indirectly the synthesis or release of prostaglandin F2a (PGF2a), which may produce the strong and frequent uterine contractions and inhibit maternal recognition of pregnancy (54). During fresh embryo transfer cycle after controlled ovarian hyperstimulation or in artificial preparation cycles for frozen embryo transfer, women undergoing IVF-ET were likely to be exposed to supraphysiologic levels of estradiol, which could affect uterine contractions and negatively affect implantation. It was reported that RIF patients may experience more uterine contractions (55). Patients with RIF experienced more hormone stimulations and more instrumental operations, such as ovarian stimulation, constantly transvaginal ultrasound supervision, transvaginal oocyte retrieval, embryo transfer or even hysteroscopy which may lead to a hyperactivated autocrine/paracrine OT/OTR system in the endometrial epithelium that can result in the high level of serum OT and PGF2α and thereby to high uterine contractions (38). This provided some level of support for the application of atosiban in patients with RIF. Correspondingly, there may be a reduced pregnancy rate among women who experience frequent uterine contractions. Therefore, drugs or treatments to decrease uterine contractions around embryo transfer are becoming more appealing options for improving pregnancy outcomes of RIF patients. Atosiban, as a combined oxytocin/vasopressin V1A antagonist, could be a choice to reduce uterine contractions. Apart from the reduction in uterine contractions, atosiban has been found to prevent early luteal regression and embryonic loss, and inhibit contractions and inflammation, by inhibiting the endometrial production of PGF2a (56, 57). Another significant effect of atosiban may be that it reversed the consequences of high estradiol and oxytocin on endometrial receptivity parameters (58). Its safety and few side effects have been evidenced in trustworthy documents in related studies (59). The phenomenon of improved pregnancy rates in patients with RIF who received atosiban could be attributed to its effects on uterine contractility and beneficial effects on endometrial receptivity.

This study provided documented evidence for the use of atosiban in cases of RIF and the potential indication for ET by comparing the pregnancy outcomes of RIF patients treated with atosiban and control. It also showed that the application of atosiban around embryo transfer could improve the pregnancy outcomes of patients with RIF.

This was the most up-to-date review, which included a large sample of patients with RIF on this subject. Both observational and randomized controlled trials confirmed the increased risk of pregnancy caused by atosiban. However, there were some potential limitations in this meta-analysis. First, the therapeutic schedules, including ovulation induction protocol, embryo transfer protocol and luteal support regimen differed among patients undergoing IVF-ET. In addition, confounding factors included the different class and number of transferred embryos. Secondly, the included studies contained various types of study design, such as randomized controlled trial and cohort study. Also, in some of the included studies, blinding was not applied. As a result, biases in implementation and measurement were unavoidable. Thirdly, the patients enrolled in the included studies represented a wide range in age, from 20 to 40. The lack of sufficient data on age in included studies meant that age specific analyses could not be performed. It has previously been indicated that the addition of atosiban to FET cycles did not decrease uterine peristalsis, but may be beneficial to the group of advanced age (60). It will be more accurate and objective if the clinical trial can be carried out by age groups.

## 5 Conclusion

In conclusion, the application of atosiban around the time of ET could increase the implantation rates, positive pregnancy test rates, clinical pregnancy rates, and live birth rates for RIF patients undergoing IVF-ET and had no effect on the rates of miscarriage, multiple pregnancy and ectopic pregnancy when compared to control groups. To investigate the efficacy of atosiban during ET in ART for RIF in more depth, further large, well-designed, prospective randomized placebo-controlled trials with large numbers of patients grouped by age and reporting on live births and adverse clinical outcomes should be conducted.

## Author contributions

RW: Research scheme design, data collection and analysis, manuscript writing. HH: Database searching, research extracting and evaluating. GX and YT: Research design and guidance. All authors contributed to the article and approved the submitted version. 

## Supplementary material

Supplementary Figure 1 Forest plots of positive pregnancy test rate.

Supplementary Figure 2 Forest plots of clinical pregnancy rate.

Supplementary Figure 3 Forest plots of live birth rate.

Supplementary Figure 4 Forest plots of implantation rate.

Supplementary Figure 5 Forest plots of miscarriage rate.

Supplementary Figure 6 Forest plots of multiple pregnancy rate.

Supplementary Figure 7 Forest plots of ectopic pregnancy rate.
