# Supplementary figures and images for: Efficacy of atosiban for repeated embryo implantation failure: A systematic review and meta-analysis
Source: Front Endocrinol (Lausanne). 2023 Mar 23;14:1161707. doi: 10.3389/fendo.2023.1161707 (PMC10076890; doi:10.3389/fendo.2023.1161707)

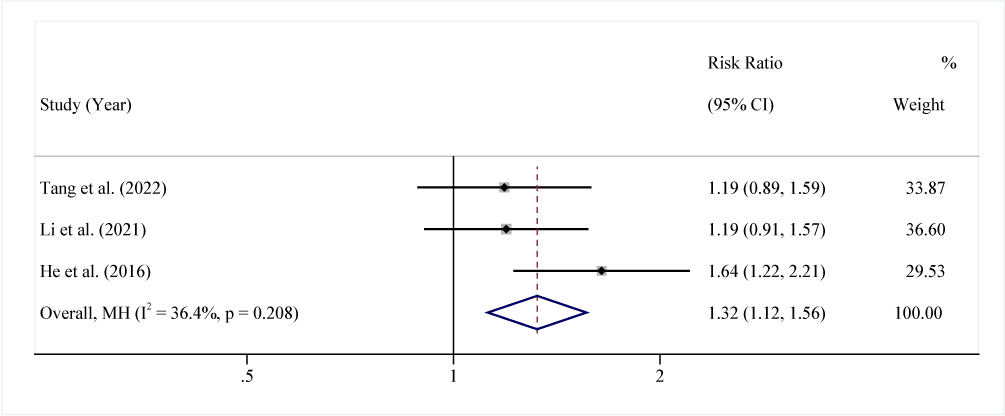

Supplement: Supplementary Figure 1 — Forest plots of positive pregnancy test rate. [file Image_1.tif]

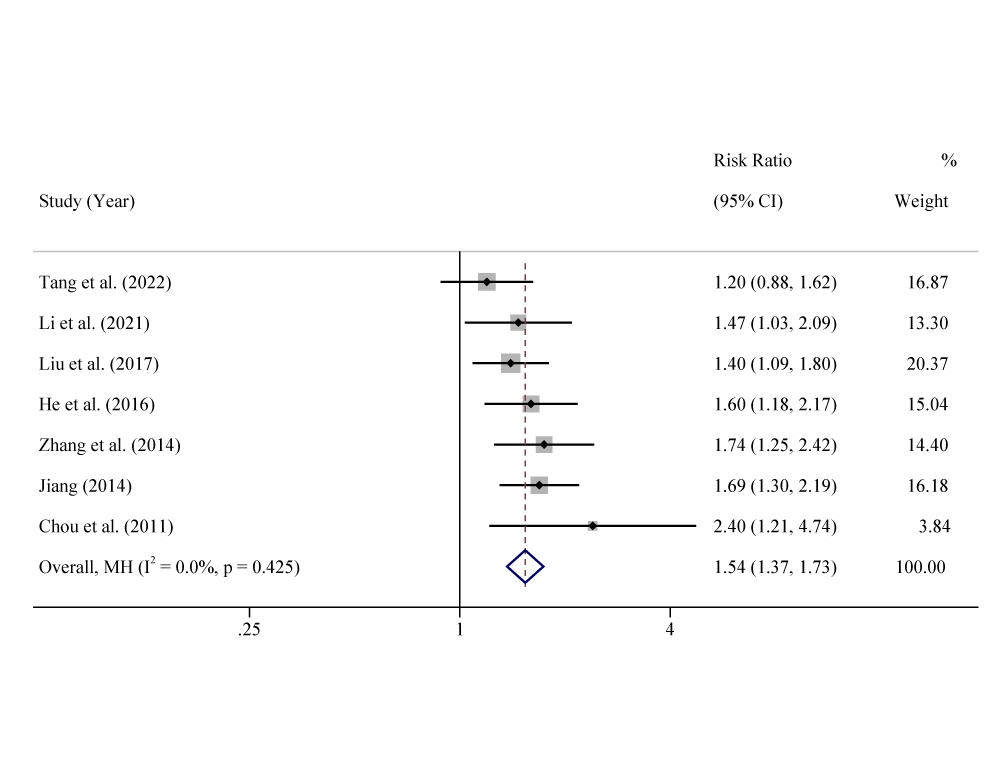

Supplement: Supplementary Figure 2 — Forest plots of clinical pregnancy rate. [file Image_2.tif]

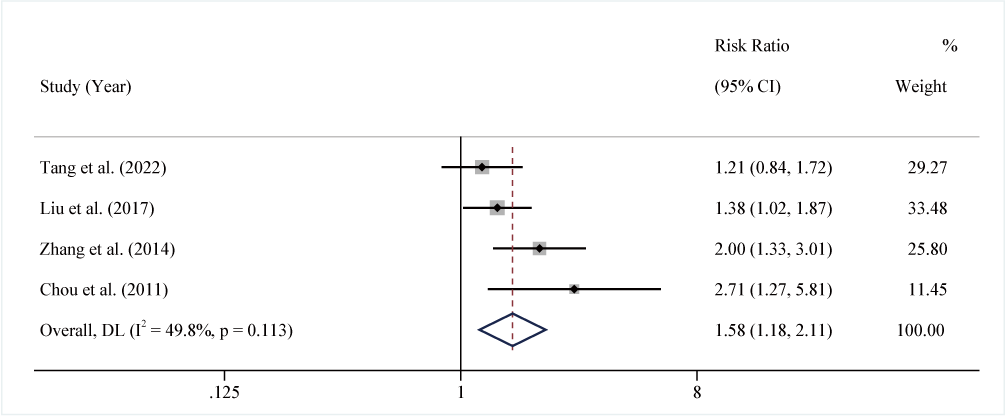

Supplement: Supplementary Figure 3 — Forest plots of live birth rate. [file Image_3.tif]

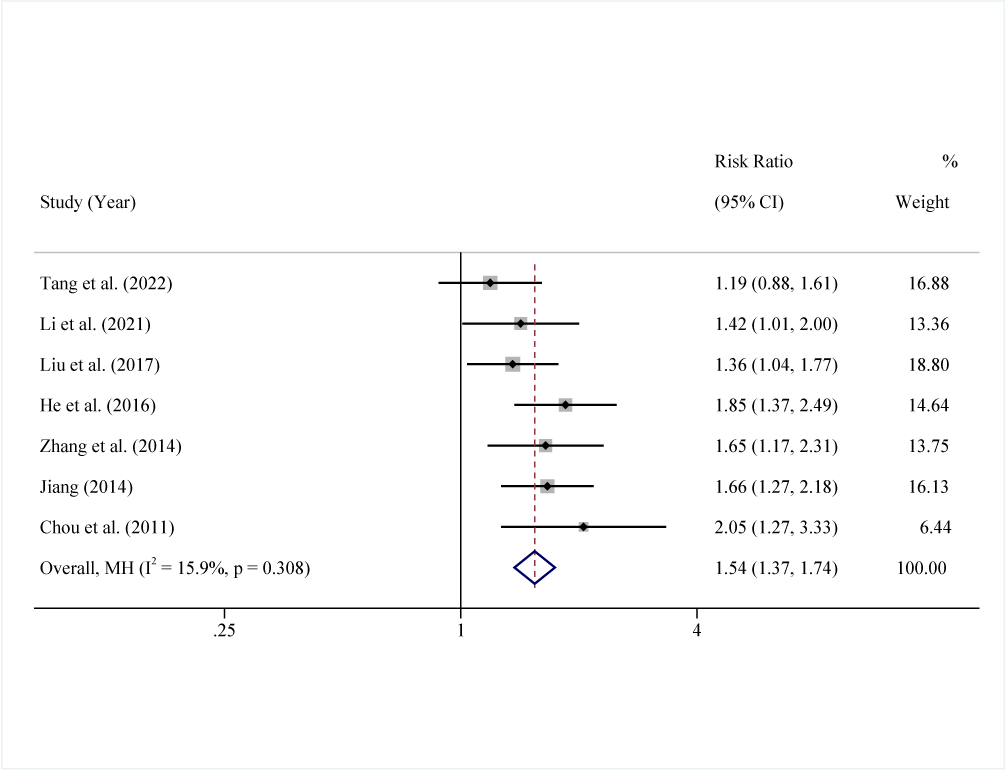

Supplement: Supplementary Figure 4 — Forest plots of implantation rate. [file Image_4.tif]

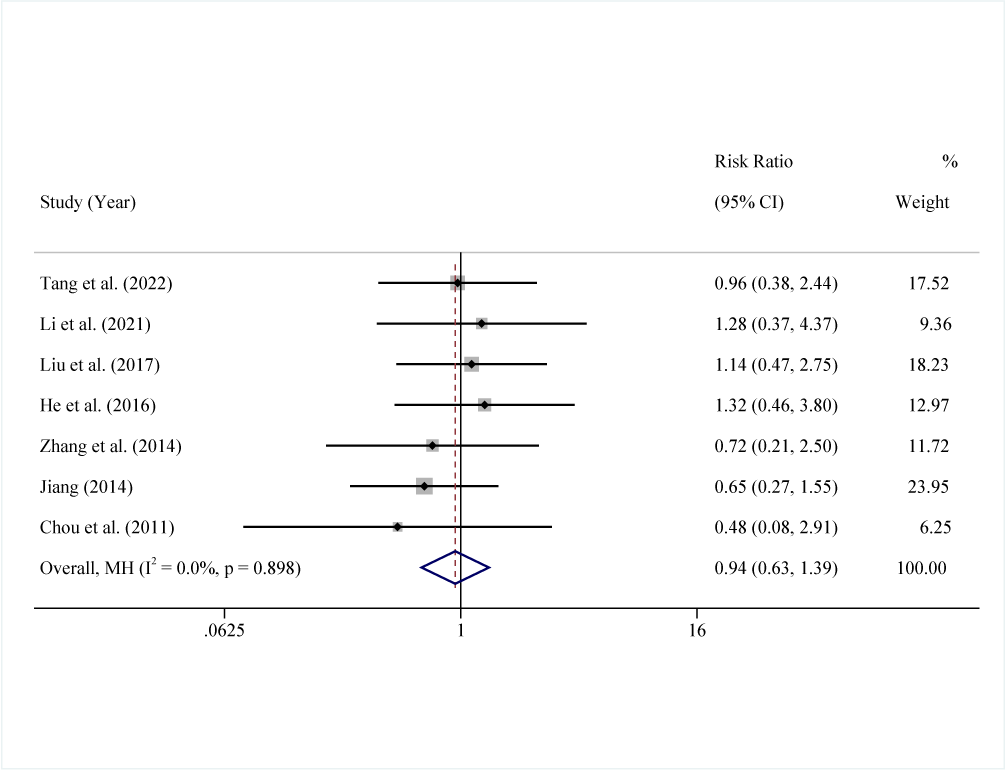

Supplement: Supplementary Figure 5 — Forest plots of miscarriage rate. [file Image_5.tif]

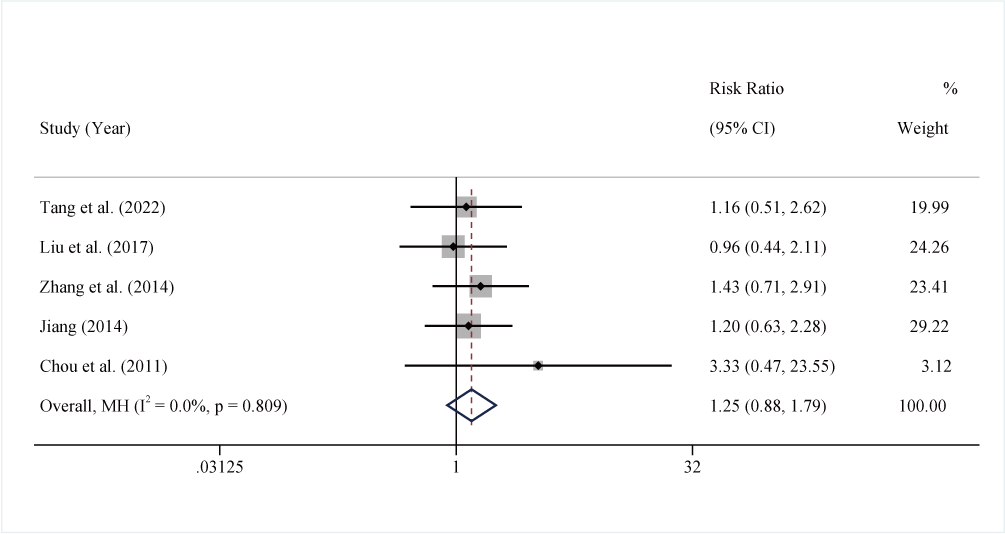

Supplement: Supplementary Figure 6 — Forest plots of multiple pregnancy rate. [file Image_6.tif]

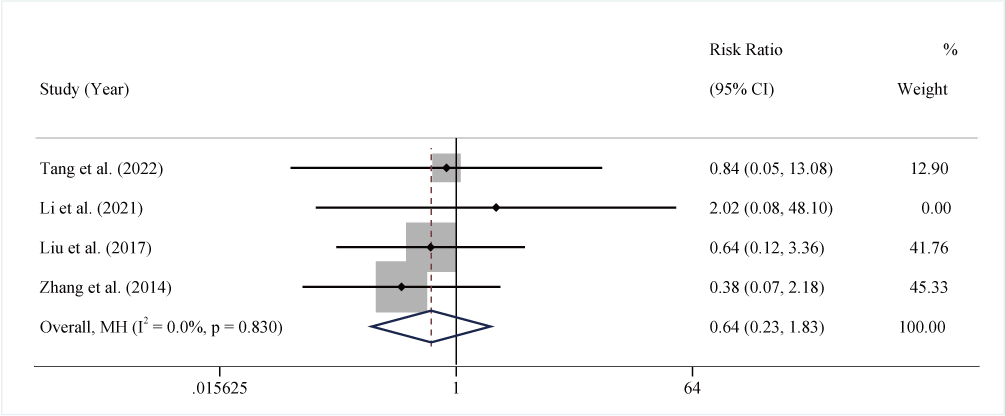

Supplement: Supplementary Figure 7 — Forest plots of ectopic pregnancy rate. [file Image_7.tif]
